# Clinical Prediction Tool to Assess the Likelihood of a Positive SARS-Cov-2 (COVID-19) Polymerase Chain Reaction Test in Patients with Flu-like Symptoms

**DOI:** 10.5811/westjem.2020.12.49200

**Published:** 2021-03-24

**Authors:** Barbara A. Lara, Francisco Torres, Patricia Holger, Claudia Perales, Sofia Basauri, Hans Clausdorff, Ernesto Escobedo, Fernando Saldias, Stuart Swadron, Pablo Aguilera

**Affiliations:** * Pontificia Universidad Católica de Chile, Section of Emergency Medicine, Santiago, Chile; † Pontificia Universidad Católica de Chile, Department of Respiratory Diseases, Santiago, Chile; ‡ Keck School of Medicine, Department of Emergency Medicine, University of Southern California, Los Angeles, California

## Abstract

**Introduction:**

The clinical presentation of coronavirus disease 2019 (COVID-19) overlaps with many other common cold and influenza viruses. Identifying patients with a higher probability of infection becomes crucial in settings with limited access to testing. We developed a prediction instrument to assess the likelihood of a positive polymerase chain reaction (PCR) test, based solely on clinical variables that can be determined within the time frame of an emergency department (ED) patient encounter.

**Methods:**

We derived and prospectively validated a model to predict SARS-CoV-2 PCR positivity in patients visiting the ED with symptoms consistent with the disease.

**Results:**

Our model was based on 617 ED visits. In the derivation cohort, the median age was 36 years, 43% were men, and 9% had a positive result. The median time to testing from the onset of initial symptoms was four days (interquartile range [IQR]: 2–5 days, range 0–23 days), and 91% of all patients were discharged home. The final model based on a multivariable logistic regression included a history of close contact (adjusted odds ratio [AOR] 2.47, 95% confidence interval [CI], 1.29–4.7); fever (AOR 3.63, 95% CI, 1.931–6.85); anosmia or dysgeusia (AOR 9.7, 95% CI, 2.72–34.5); headache (AOR 1.95, 95% CI, 1.06–3.58), myalgia (AOR 2.6, 95% CI, 1.39–4.89); and dry cough (AOR 1.93, 95% CI, 1.02–3.64). The area under the curve (AUC) from the derivation cohort was 0.79 (95% CI, 0.73–0.85) and AUC 0.7 (95% CI, 0.61–0.75) in the validation cohort (N = 379).

**Conclusion:**

We developed and validated a clinical tool to predict SARS-CoV-2 PCR positivity in patients presenting to the ED to assist with patient disposition in environments where COVID-19 tests or timely results are not readily available.

## INTRODUCTION

The novel coronavirus disease 2019 (COVID-19) pandemic poses a major threat to global health.^1^ Despite public health efforts to contain its rapid spread, outbreaks have led to emergency department (ED) crowding, a strain on hospital resources, and a shortage in testing capacity. In the absence of effective prophylaxis or a vaccine, the most efficient containment strategy is interrupting transmission through rapid identification and isolation of infected patients.^2^

Given that the clinical presentation of COVID-19 overlaps with many other common cold and influenza viruses, identifying patients with a higher probability of infection becomes crucial in settings with limited access to testing.^3^,^4^ The most commonly reported symptoms are fever, myalgia, fatigue, headache, dry cough, and dyspnea, whereas less frequent symptoms include rhinorrhea, sore throat, diarrhea, chest tightness, anosmia, dysgeusia, and hemoptysis.^4^–^6^ Reported laboratory findings include leukopenia, leukocytosis, lymphopenia, high lactate dehydrogenase, and a host of elevated inflammatory markers.^7^due to the novel severe acute respiratory syndrome coronavirus 2 (SARS-CoV-2 Imaging findings include patchy, ground-glass infiltrates on chest radiograph (CXR) and computed tomography.^4^,^8^

The turnaround time for the SARS-CoV-2 polymerase chain reaction (PCR) test in our ED and in the vast majority of Chilean centers is at least 48 hours and up to 10 days. Moreover, shortages of tests and reagents limit the number of tests that we can perform. To facilitate the disposition of our patients without the benefit of PCR testing results, we developed a prediction instrument to assess the likelihood of a positive PCR test, based solely on clinical variables that can be determined within the time frame of an ED patient encounter.

## METHODS

We conducted a two-phase observational study involving patients visiting the ED with symptoms consistent with COVID-19 who were tested with a SARS-CoV-2 PCR test over two consecutive months (March and April 2020) during the beginning of the pandemic in Chile. Data were collected in an urban, academic hospital with 380 beds and 32 adult intensive care unit beds, expanded to 75 critical care beds during the pandemic. The ED receives an average of 40,000 adult patient visits per year.

In the first phase, during March 2020, data were collected retrospectively from the national COVID-19 notification form required for all patients tested for COVID-19 in our ED. This was complemented with vital signs, and laboratory and imaging results from the patient health record. We used the information collected to derive a prediction instrument for COVID-19 diagnosis. In the second phase, conducted over the subsequent four weeks, we prospectively applied a new data collection form to validate our instrument. In this phase, all patients who were tested for COVID 19 were included. The decision to order the test was at physician discretion. Clinicians were blinded to the study results of the first phase and completed an extended form including all variables tested in the first phase.

Cases were individuals who had a positive SARS-CoV-2 PCR result, and controls were those with a negative test. We excluded patients younger than 18 years old, pregnant women, patients who returned for a second visit to the ED in the following month, those who had already been tested for COVID-19 prior to the ED visit, those with an indeterminate test result, and those for whom less than 30% of the data required for the derivation set was available. We collected and managed study data using Research Electronic Data Capture tools hosted at Pontificia Universidad Católica de Chile (REDCap Consortium, Vanderbilt University, Nashville, TN). This study was approved under a waiver of informed consent by our institutional review board.

**Population Health Research Capsule**

What do we already know about this issue?

*The clinical presentation of coronavirus disease 2019 (COVID-19) overlaps with many other common cold and influenza viruses.*

What was the research question?

*Can we predict COVID-19 test positivity based on clinical variables?*

What was the major finding of the study?

*Anosmia, dysgeusia, fever, headache, myalgia, dry cough, and history of close contact are good predictors of COVID-19 diagnosis.*

How does this improve population health?

*This group of symptoms could be used to assist with patient disposition in environments where COVID-19 tests or timely results are not readily available.*

### Variables

We included in the analysis demographic information; general symptoms (including myalgias, fatigue, quantified fever at home, headache); respiratory symptoms (including rhinorrhea, sore throat, dry, and productive cough, dyspnea); gastrointestinal symptoms (including anorexia, vomiting, diarrhea, nausea, and abdominal pain); and anosmia or dysgeusia. Comorbidities, tobacco and drug use, vaping, medications, influenza vaccination history, and other epidemiologically relevant data such as travel to countries with outbreaks and close contacts with confirmed cases of COVID-19 were also collected and analyzed. We used the Chilean national health definition of “close contact” (high-risk exposure), which includes patients who were exposed to another person with a positive test starting two days before symptoms onset: a) for at least 15 minutes at less than three feet distance without the use of a mask; b) patients who shared a closed space (such as a room or office) for more than one hour without a mask; and c) patients who slept in the same room or lived together in the same house. We defined “febrile” as a self-reported temperature at home or axillary temperature in the ED ≥ 38°C. Time from symptoms onset, vital signs, laboratory, and imaging results were also included in the analysis.

### Statistical Analysis

For the derivation cohort, we examined group differences using chi-square and t-tests and considered a *P*-value <0.05 for statistical significance. Because most ambulatory patients in the ED do not receive laboratory or imaging studies, we planned to create two separate models, the first (full model) based only on clinical data (demographics, comorbidities, signs and symptoms, and vital signs) and a second (restricted model) incorporating laboratory and imaging results. To develop both models, we used the retrospective derivation set and fit the model using logistic regression with a stepwise, purposeful forward selection of variables. We first selected variables that were statistically significant with a *P*-value <0.1 and added them one by one in the multivariable logistic regression model (full model). We then compared the full vs restricted model with a likelihood ratio test and kept the variables that added statistical value to the model. At the end of this process we added back all variables that were not statistically significant in the univariate analysis and kept those that improved the model. After checking for collinearity, we retained or dropped variables depending on their clinical relevance as well as their statistical influence in the main effect variable model.

We assessed the performance of the final model using the Hosmer-Lemeshow goodness of fit test and its discriminatory performance by an area under the ROC curve (AUROC). We tested the model in the prospective cohort using the same performance parameters. We used Stata statistical software (StataCorp, College Station, TX) for all analyses. Finally, we created a score based on the main effect model to make it more suitable for clinical practice. We described its discriminatory power for several cut-points with likelihood ratios and the rate of PCR COVID-19 positivity by categories.

## RESULTS

For the derivation cohort, we assessed the charts of 682 ED visits that met inclusion criteria. We excluded 61 patients under 18 years old, five because of missing data, two due to a missing PCR result, and a single patient because the PCR COVID-19 result was indeterminate. We extracted data on the remaining 617 patients. Of those patients, 43% (N = 262) were men with a median age of 36 years (interquartile range [IQR]: 29–49, range 19–96 years). In the prospective cohort of 379 consecutive patients, 46% were men with a median age of 39 years (IQR: 30–53, range 12 – 98 years). The main demographic and clinical characteristics for the derivation and validation cohorts are shown in Table 1. The rate of COVID-19 positive tests was 9% (N = 58) in the derivation cohort and 18% (N = 69) in the validation cohort (*P*<0.001). For both groups, the median time to testing from the onset of initial symptoms was days (IQR: 2–5 days, range 1–23 days), and 91% of all patients were discharged home. The rate of COVID-19 positive patients was 12% for outpatients and 13% for inpatients (*P*>0.05). Only two patients had Mapuche ancestry (an indigenous Chilean population). Both tested negative for COVID-19 and were discharged home.

### Univariate Analysis

In the univariate analysis, the clinical variables that had the highest positive likelihood ratios were anosmia or dysgeusia, fever, history of close contact, use of angiotensin-converting enzyme (ACE) inhibitors, lack of dyspnea, oxygen saturation below 95% on room air, and an abnormal chest CXR (Table 2).

### Prediction Model/Development Set

Variables that were statistically significant at the *P*< 0.1 level are shown in Table 2. Age and gender were not associated with a positive test result. The only comorbidity positively associated with a positive test was hypertension and the use of ACE inhibitors. A history of close contact with a person who had tested positive was also significant. Symptoms such as fever, myalgias, headache, dry cough, anosmia or dysgeusia, and lack of shortness of breath were predictors of a positive PCR COVID-19 result. Oxygen saturation lower than 95% on room air was also found to be predictive. Among tests and imaging results, cases were more likely to have an abnormal CXR than controls.

Although use of ACE inhibitors improved the final model numerically, we decided to drop that variable for lack of robust physiologic evidence that supported that association (adjusted odds ratio [AOR] 2.5, *P* = 0.28). We also excluded oxygen saturation below 95% despite its statistical significance in the multivariable model (AOR 2.36, *P* = 0.042) because we considered that abnormal vital sign as a marker of higher acuity and determinant of hospital admission and further testing rather than a predictor of etiology. Table 3 shows the multivariable logistic regression coefficients, Wald test, and odds ratio for each of the predictor variables for the final model.

### Fit and Discrimination Power Model Assessment

The final logistic regression model for clinical data and its covariates is shown in Table 3. By internal bootstrap validation, the mean AUC based on data from the development cohort was 0.79 (95% confidence interval [CI], 0.73–0.85). When assessing the fit of the model, the Hosmer-Lemeshow test demonstrated *P*>0.05 denoting good model fit. Because of the small number of patients with available laboratory and imaging data, we were not able to create a model with these variables as planned. We report their univariate analysis in Table 2.

### Prospective Validation

The discrimination power for the clinical model in the validation cohort had an AUC 0.70 (95% CI, 0.61–0.75) and a good fit (Hosmer and Lemeshow *P*-value = 0.943).

### Prediction Tool

To make this information useful for clinical practice, we created a weighted score (Table 4). For each point over 0, the rate of PCR SARS-CoV-2 positivity increases significantly from 4% in the subgroup with 0 points to 80% for those with 8 points. For cut-points at 4, 10 and 14 points the likelihood ratios were 1.4, 4.6 and 10, respectively. Thus, we further classified the score into four categories. Its PCR COVID-19 positivity is shown in Table 5.

## DISCUSSION

The most frequently reported symptoms among COVID-19 patients during their ED visit were dry cough, myalgias, and fever. Despite anosmia and dysgeusia not being as frequently reported as in other series (9% in our retrospective cohort and 20% in the prospective cohort vs up to 50% in other reports), they were highly correlated with positive PCR test (likelihood ratio 5.5).^6^,^9^ We believe that the higher frequency observed in our validation cohort is consistent with an increasing awareness of these symptoms in both medical and lay communities over time as the pandemic unfolded in Chile. The same phenomenon might explain the lower prevalence of gastrointestinal symptoms in our cohort as compared with international data.

Studies to date have focused on confirmed hospitalized cases and on prognostic factors for adverse outcomes during hospital stay.^10^–^13^ Fewer studies have described the characteristics of patients assessed for suspicion of COVID-19 in the ED and other ambulatory settings. Moreover, the latest have been reliant on laboratory and imaging data, while a few models incorporating signs and symptoms have not been prospectively validated.^14^–^19^

Our model includes symptoms and risk factors for COVID-19 infection that have been described as frequent among patients with COVID-19 infection.^7^ However, none of these alone had a likelihood ratio that would allow crossing the testing threshold, except for anosmia or dysgeusia and an abnormal CXR. In our study, however, anosmia or dysgeusia were not frequently reported. Moreover, the influence of CXR results may have been exaggerated in our study because imaging is not frequently performed in low-acuity patients in our ED and was obtained only at emergency physician discretion during the study period.

Whereas individual symptoms, signs, and results of readily available testing may lack accuracy in predicting infection with COVID-19, in aggregate they might inform decision-making with respect to the utilization of PCR testing and ED discharge planning. Although models that include imaging or laboratory findings perform better, our clinical-only model could be very helpful for a low-acuity cohort of patients who under normal (pre-pandemic) circumstances would not receive any testing in the ED. In this group of patients, it might help us improve throughput by reducing the need for laboratory testing and imaging. Moreover, such a tool could be key for decision-making in periods of the pandemic when testing was unavailable or very limited.

Because patients in the derivation cohort presented early in the course of the pandemic in Chile, we expected a much higher rate of COVID-19 positive patients in the validation cohort. This higher rate of COVID-19 positive patients in the validation cohort might explain the decrease in the discriminatory power of the model. However, the increasing rate of positive cases for each cut-point of the simplified score in both cohorts is consistent.

## LIMITATIONS

We identify four potential limitations. First, we included all patients visiting the ED who had a COVID-19 test performed to be able to extrapolate the findings to a broader cohort, including those who were admitted. However, this strategy might also have blurred the true relationship between the outcome and the variables, affecting its performance. Children are more likely to be asymptomatic than adults, while elderly and admitted patients who visit the ED with more severe disease may report only the severe symptoms such as dyspnea and chest discomfort. This might contribute to a diminished accuracy of the score for middle-aged, ambulatory patients.

Second, we used a unique SARS-CoV-2 PCR as the gold standard at the time of ED visit, which has been criticized for its limited sensitivity. To investigate the impact of potential false negative tests on our results, we calculated the rate of negative to positive conversion in the proportion of patients who were tested again during the following month. About 1% of admitted and ambulatory patients converted at follow-up. Although the minority of all ambulatory COVID-19 negative patients had a repeated test, we believe that because all our patients had symptoms at the time of testing, we probably tested them at the peak of the sensitivity curve and thus the rate of false negative tests was not significant. Moreover, more than 50% of patients with repeated testing in the ambulatory setting that turned positive were tested more than 14 days after the initial encounter, and presented with new symptoms, suggesting a new infection rather than reflecting an initial false positive.

Third, although we aimed to include imaging and laboratory results as part of a second model, we failed to include these variables because of the small sample size and did not externally validate the model in other clinical settings outside our institution. Lastly, our study was performed during a period of time where SARS-CoV-2 was the predominant circulating virus in our community. Thus, a validation during influenza season would be required to extrapolate these results to a time period when other seasonal viruses start to circulate.

## CONCLUSION

Our clinical prediction instrument demonstrated the ability to predict a positive SARS-CoV-2 PCR in patients presenting to the ED with flu-like or cold symptoms with moderate accuracy. Such a tool could be used to assist with patient disposition in environments where COVID-19 tests or timely results are not readily available.

## Figures and Tables

**Table 1:** Characteristics of derivation and validation cohorts.

Characteristics	Derivation N = 617 n (%)	Validation N = 379 n (%)	All patients N = 996 n (%)
Demographics			
Age (median, years)	40	42	41
Male gender	263(43)	176(46)	439(44)
Ethnicity (Mapuche)	1(0.2)	1(0.2)	2(0.2)
Comorbidities			
Hypertension	86(14)	69 (18)	155(16)
Diabetes	27(4)	25(7)	52(5)
Obesity	10(2)	38(10)	48(5)
Chronic kidney disease	5(1)	5(1)	10(1)
Coronary cardiopathy	12(2)	5(1)	17(2)
Heart failure	11(2)	5(1)	16(2)
Immunosuppression	24(4)	13(3)	37(4)
HIV	7(1)	6(2)	13(1)
Active cancer	14(2)	4(1)	18(2)
ACE inhibitors	63(10)	47(12)	110(11)
Asthma	40(6)	25(7)	65(7)
COPD	10(2)	4(1)	14(1)
Pulmonary fibrosis	3(0)	1(0)	4(0)
Smoking	61(10)	64(17)	125(13)
Marijuana use	9(1)	14(4)	23(2)
Disposition			
Discharged home	560(91)	343(91)	903(91)
Symptoms			
Fever	169(27)	94(25)	263(26)
Myalgias	240(39)	178(47)	418(42)
Headache	172(28)	175(46)	347(35)
Anosmia or dysgeusia	14(2)	32(8)	46(5)
Dyspnea	92(15)	50(13)	142(14)
Malaise	153(25)	131(35)	284(289)
Rhinorrhea	182(30)	94(25)	276(28)
Sore throat	337(55)	166(44)	503(51)
Dry cough	323 (52)	149(39)	472(47)
Sputum	29(5)	27(7)	56(6)
Nausea	17(3)	24(6)	41(4)
Vomiting	16(3)	13(3)	29(3)
Diarrhea	53(9)	66(17)	119(12)
Abdominal pain	28(5)	37(10)	65(7)
Vital signs			
Heart rate (bpm), mean±SD	87±17	87±18	87±17
SBP (mmHg), mean±SD	131±32	136±20	134±28
DBP (mmHg), mean±SD	79±12	80±12	80±12
Oxygen Sat (%), mean±SD	97±2.9	97±2	97±2.7
RR (rpm), mean±SD	21±7	20±4	21±6
Temperature* (C°), mean±SD	36.6±0.7	36.5±0.7	36.5±0.7
PCR COVID-19			
Positive	58(9)	69(18)	127(13)

* Temperature= Axillary temperature.

*HIV*, human immunodeficiency virus; *ACE*, angiotensin-converting-enzyme; *COPD*, chronic obstructive pulmonary disease; *bpm*, beats per minute; *SD*, standard deviation; *SBP*, systolic blood pressure; *mmHg*, millimeters of mercury; *DBP*, diastolic blood pressure; *RR*, respiratory rate; *rpm*, respirations per minute; *C°*, Celsius; *PCR*, polymerase chain reaction; *COVID-19*, coronavirus disease 2019.

**Table 2:** Univariate analysis of clinical variables as predictors of positive PCR COVID-19.

Variables	OR	95% CI	P-value	Sensitivity	Specificity	LR
Demographics						
Age (per year)	1.00	0.99 – 1.02	0.362			
Male gender	1.39	0.81 – 2.39	0.228	0.50	0.58	1.19
Other						
Close contact†	2.01	1.14–3.56	0.016	0.36	0.78	1.64
ACE inhibitor use	1.98	0.95 – 4.1	0.068	0.17	91	1.81
Symptom onset (days)	1.64	0.97 – 1.1	0.276			
Symptom onset ≥ 4 days	2.45	0.94 – 2.9	0.083	0.45	0.66	1.45
Obesity		0.5 – 11.8	0.262	0.03	0.98	2.4
General symptoms						
Malaise	0.96	0.51 – 1.80	0.903	0.24	0.75	0.97
Fever at home	2.99	1.72 – 5.18	<0.001	0.50	0.75	1.99
Febrile*	2.90	1.69 – 5.08	<0.001	0.50	0.74	1.99
Myalgias	3.08	1.76 – 5.42	<0.001	0.64	0.64	1.75
Headache	1.96	1.12 – 3.41	0.018	0.41	0.74	1.56
Anosmia or dysgeusia	5.76	1.86 – 17.82	0.002	0.09	0.98	5.35
Respiratory symptoms						
Rhinorrhea	0.81	0.44 – 1.51	0.524	0.26	0.70	0.86
Dry cough	1.82	1.03 – 3.21	0.037	0.66	0.49	1.28
Productive cough	0.33	0.04 – 2.49	0.292	0.01	0.95	0.34
Sore throat	0.69	0.40 – 1.20	0.197	0.47	0.45	0.83
Dyspnea	0.28	0.08 – 0.94	0.039	0.05	0.84	0.3
Gastrointestinal symptoms						
Nausea	2.12	0.59 – 7.61	0.248	0.05	0.98	2.0
Vomiting	1.39	0.30 – 6.27	0.668	0.03	0.98	1.37
Diarrhea	1.25	0.51 – 3.08	0.617	0.10	0.91	1.23
Abdominal pain	1.65	0.55 – 4.93	0.369	0.07	0.96	1.60
Vital signs						
Temperature ≥ 38°C	1.78	0.71 – 4.41	0.227	0.10	0.94	1.7
Heart rate > 100 bpm	1.38	0.71 – 2.67	0.33	0.24	0.81	1.29
SBP < 100 mmHg	1	-	-	0	0.98	0.00
DBP < 60 mmHg	1.4	0.70 – 2.6	0.359	0.01	0.94	0.29
RR > 20 rpm	1	-	-	0	1	-
Oxygen Sat < 95%	2.16	1.03 – 4.55	0.041	0.17	0.91	1.96
Imaging**						
Abnormal chest radiograph	4.3	1.22–15.3	0.023	0.67	0.68	2.11
Laboratory***						
Leukocytes > 12,000	0.17	0.02 – 1.5	0.117	0.12	0.55	0.28
LDH > 200	3.1	0.3 – 27	0.31	0.85	0.34	1.3

† Close contact: defined as having been exposed to another person with a positive test starting two days before symptoms developed for at least 15 minutes, contact with another person at less than three feet distance without the use of a mask, sharing a closed space (such as a room or office) for more than one hour without a mask, slept in the same apartment or lived together in the same house.

* Febrile was defined as self-reported quantified fever (axillary temperature ≥38°C) at home or had an axillary temperature ≥ 38°C at any time in the emergency department (ED).

** Based on 207 observations that had a chest radiograph ordered in the ED.

*** Based on 78 observations that had laboratory test ordered.

*PCR*, polymerase chain reaction; *COVID-19*, coronavirus disease 2019; *OR*, odds ratio, *CI*, confidence interval; *LR*, likelihood ratio; °*C*, degrees Celsius; *bpm*, beats per minute; *SBP*, systolic blood pressure; *mmHg*, millimeters of mercury; *DBP*, diastolic blood pressure; *RR*, respiratory rate; *rpm*, respirations per minute.

**Table 3:** Final prediction model.

Variable	Coefficient	OR (95%CI)	*P*
Anosmia or dysgeusia	2.3	9.7 (2.72 – 34.5)	<0.001
Febrile	1.3	3.63 (1.93 – 6.85)	<0.001
Myalgias	0.95	2.6 (1.39 – 4.89)	0.003
Close contact	0.90	2.47 (1.29 – 4.7)	0.006
Headache	0.67	1.95 (1.06– 3.58)	0.031
Dry cough	0.66	1.93 (1.02 – 3.64)	0.040

N = 617, LR chi^2^ = 56.94, Prob > chi^2^ = < 0.001, Pseudo R^2^ = 0.15.

*OR*, odds ratio; *CI*, confidence interval.

**Table 4:** Final scores.

Variables	Points
Anosmia or dysgeusia	8
Febrile	4
Myalgias	3
Close contact	3
Headache	2
Dry cough	2

**Table 5:** PCR COVID-19 positivity by score category.

Score categories	All, n	PCR SARS-CoV-2 Positive, n (%)
Low risk (0 – 4 pts)	429	24(5.3)
Intermediate risk (5 – 9 pts)	433	59(14)
High risk (10 – 13 pts)	78	25(32)
Very high risk (≥14 pts)	32	19(59)
All patients	996	127 (13)

*PCR*, polymerase chain reaction; *COVID-19*, coronavirus disease 2019.
